# A quality improvement pilot assessment of the safety and associated outcomes of a viable cryopreserved umbilical tissue allograft as an adjunct surgical wrap in peroneus brevis tendon repair

**DOI:** 10.1097/MD.0000000000013662

**Published:** 2018-12-21

**Authors:** Kyle W. Sundblad, Elisabet K. Tassis

**Affiliations:** a Macomb Foot, Ankle and Wound Care, Sterling Heights, MI; b Osiris Therapeutics, Columbia, MD.

**Keywords:** allograft, peroneal tendon, rupture, stravix, viable cryopreserved umbilical tissue

## Abstract

Surgical tendon repairs of the lower extremity are frequently associated with post-operative (post-op) risks that result in poor patient outcomes. Initially, increased pain levels may contribute to extended post-op rehabilitation while the development of tissue adhesions and fibrosis limit long-term functionality through reduced range of motion. Several surgical methods describing incorporation of various augmentative graft materials in tendon repair exist. However, reports demonstrating technique and both short- and long-term patient outcomes are lacking. Recently, advances in tissue preservation technology have led to the commercialization of human placental allografts. Of these available allogeneic biomaterials, the components found in human placental membranes may provide anti-inflammatory, antimicrobial, anti-adhesive, and antifibrotic properties to benefit surgical outcomes.

Here, the authors introduce and technically describe the use of a viable cryopreserved umbilical tissue (vCUT) (Stravix, Osiris Therapeutics, Inc., Columbia, MD) as a complementary surgical wrap in primary tendon repair, with particular focus on the peroneus brevis. A pilot study was undertaken to assess the safety and potential for secondary rehabilitative outcomes associated with the use of vCUT in 5 tendon repair cases. The use of vCUT as a surgical tendon wrap was evaluated via the following primary endpoints at post-op day 7:

1. presence of erythema, tenderness, heat and/or swelling;

2. pain score;

3. patient use of narcotic medication; and

4. the development of adverse events during any point during the post-op course, defined in this study as dehiscence, confirmed infection, fluid collection or drainage.

Secondary investigative endpoints included clinical and rehabilitative outcome measures for comparative pain reduction and transition times to both controlled ankle movement (CAM) boot and normal shoe ambulation.

All patients were followed for an average of 24.15 months (range 16.75–26.5 months) after surgery. For primary safety measures, erythema, tenderness, drainage, heat, and swelling was absent in all 5 surgical sites. None of the patients required post-op use of narcotics past day 7. The potential for long-term rehabilitative improvement with adjunct use of vCUT was also demonstrated through reduced pain and reduced transition times to functional and non-assisted ambulation in normal shoewear as compared to historical controls managed without vCUT.

This surgical technique is simple and safe for patients and preliminary findings have demonstrated favorable clinical and rehabilitative outcomes over historically observed controls.

## 1 Introduction

Peroneal tendon injuries are associated with debilitating complications—such as chronic ankle instability, foot deformities, chronic pain, and bone spurs^[1]^—thus, prompt diagnosis and repair are crucial for optimizing clinical outcomes. Initially, pain levels contribute to extended lengths of post-operative (post-op) rehabilitation while development of tissue adhesions and fibrosis limit long-term functionality through reduced range of motion. In recent years, several surgical techniques and protocol algorithms for tendon repair have been proposed incorporating various augmentative graft materials.^[1–3]^ Cases of acellular allograft use in tendon repair have been presented in the literature,^[4–6]^ many soliciting unwanted inflammatory reactions,^[7–9]^ yet reports on the use or outcomes of viable cellular allografts in tendon repair are scarce.

Recent advances in tissue preservation technology have led to the development and commercialization of placental tissues, one being viable cryopreserved umbilical tissue (vCUT) (Stravix, Osiris Therapeutics, Inc., Columbia, MD). Fresh umbilical tissues are known to contain a rich extracellular matrix, growth factors, and viable cells, including mesenchymal stem cells. vCUT's unique cryopreservation method—including storage at −80° C—enables the umbilical tissues to retain the naturally occurring components and the inherent anti-inflammatory, antifibrotic, analgesic, and antimicrobial properties of fresh placental tissues.^[10]^ Through retention of these properties, surgical complications such as adhesion formation, pain and inflammation may be mitigated.

Briefly, this report details a novel surgical technique incorporating the use of vCUT as a pliable and conforming tendon wrap in tendon repair, with a particular focus on the peroneus brevis tendon. The authors, through a pilot study, explore the safety of incorporating this viable allograft in surgery and investigate the existence of any beneficial clinical outcomes occurring as a result. Pilot study outcomes are presented for evaluation alongside the detailed technical tip. The results observed, have led the authors to believe that the utilization of vCUT in tendon repair supports the body's natural tissue repair process, minimizing risks for post-op infection, adhesion formation, and fibrosis that correlates with pain reduction and expedited recovery.

## 2 Materials and methods

### 2.1 Pilot study design

This pilot assessment was conducted by a single surgeon at Macomb Foot, Ankle and Wound Care after review and approval by the internal Ethics Committee at the center. The aim of the study was to evaluate the safety of a vCUT allograft when used as an adjunct to primary surgical tendon repair. A total of 5 subjects were included in this study (1 male; 4 females; age range 19–58 years; average 41 years). Patient medical histories included vertigo, diabetes and left-side dropfoot (Table 1). All patients were given a detailed explanation of the procedure and provided written informed consent. The tendon injuries were as follows: 2 Achilles ruptures, 2 peroneus brevis ruptures and 1 tibialis posterior rupture. All patients had previously failed conservative therapy consisting of rest, activity modification and physical therapy for duration of 6 months.

**Table 1:**
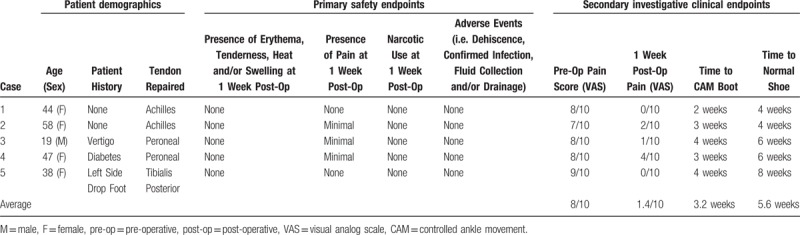
Tendon repair patient demographics and pilot study endpoint outcomes.

Primary safety endpoints of the study included:

1) presence of erythema, tenderness, heat and/or swelling at 1-week post-op confirmed via visual iatrogenic inspection,
2) presence of pain at 1-week post-op as reported by the patient,
3) narcotic use at 1-week post-op as reported by the patient, and
4) presence of any adverse events post-surgery (i.e., dehiscence, confirmed infection, fluid collection or drainage) over the entire course of follow-up.

Secondary investigative endpoints included clinical and rehabilitative outcomes of:

1) pre-operative (pre-op) pain measured with the visual analog scale (VAS),
2) one week post-op pain measured with the VAS
3) time to transition to CAM boot, and
4) time to transition to and ambulate in normal shoe.

Endpoint data were presented as mean ± standard deviation.

Post-op protocol for this study included non-weight bearing with weekly cast changes for 3 weeks. Patients were subsequently transitioned to a controlled ankle movement (CAM) boot with initiation of physical therapy and encouraged to transition to normal shoe and ambulate, as tolerated.

### 2.2 Umbilical tissue allograft preparation

**Figure 1:**
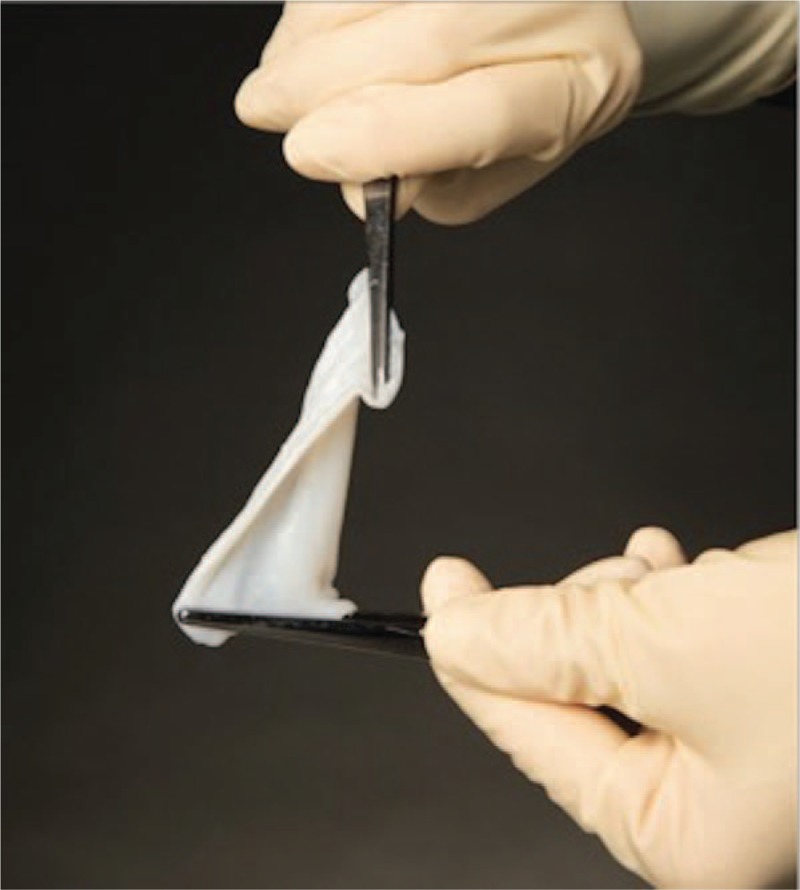
A 3 x 6 centimeter pliable and conforming vCUT allograft post thawing, ready for application at the site of surgical tendon repair. vCUT= viable cryopreserved umbilical tissue.

vCUT is delivered by the manufacturer aseptically cryopreserved in a plastic jar. To facilitate thawing at the time of required use, room temperature sterile normal saline is added to the plastic container to submerge the vCUT. Typically, after 3 to 10 minutes, ice crystals are no longer visible in the jar and preparation is considered complete. Additional tissue rinsing is not required. When ready, the pliable vCUT can be removed from the jar with forceps (Fig. 1) and conformed to the desired surgical site.

### 2.3 Surgical technique

The following surgical technique is described in detail for a peroneus brevis repair. (It is important to note, that vCUT can be applied in a similar fashion for all tendon repairs, including and not limited to Achilles, peroneal and tibialis repairs.)

The patient is placed on the operating room table in the supine position. Following induction of intravenous and local anesthesia, the affected foot is prepped and draped in the usual sterile fashion. The affected extremity is elevated approximately 60 degrees above the horizontal plane at which point a pneumatic ankle tourniquet is applied. Following exsanguination by Martin esmarch bandage, the tourniquet is rapidly inflated to 250 mmHg.

Attention is then directed towards the patient's lateral ankle at the malleolar region where approximately 8 cm modified Ollier curved incision is created along the course of the peroneal tendon posterior to lateral malleolus extending distally. The incision is deepened via sharp and blunt dissection to the level of subcutaneous tissues. All venous tributaries are electrocauterized as necessary. Care is taken to avoid trauma to the sural nerve. Dissection is deepened until the superior peroneal retinaculum is identified and incised. At this time, the sheath of the peroneus brevis and longus tendons is identified. The tendons are palpated proximally as far as the incision allows. The tendon sheath is incised in a longitudinal fashion along the course of the tendon utilizing a sharp #15 blade.

**Figure 2:**
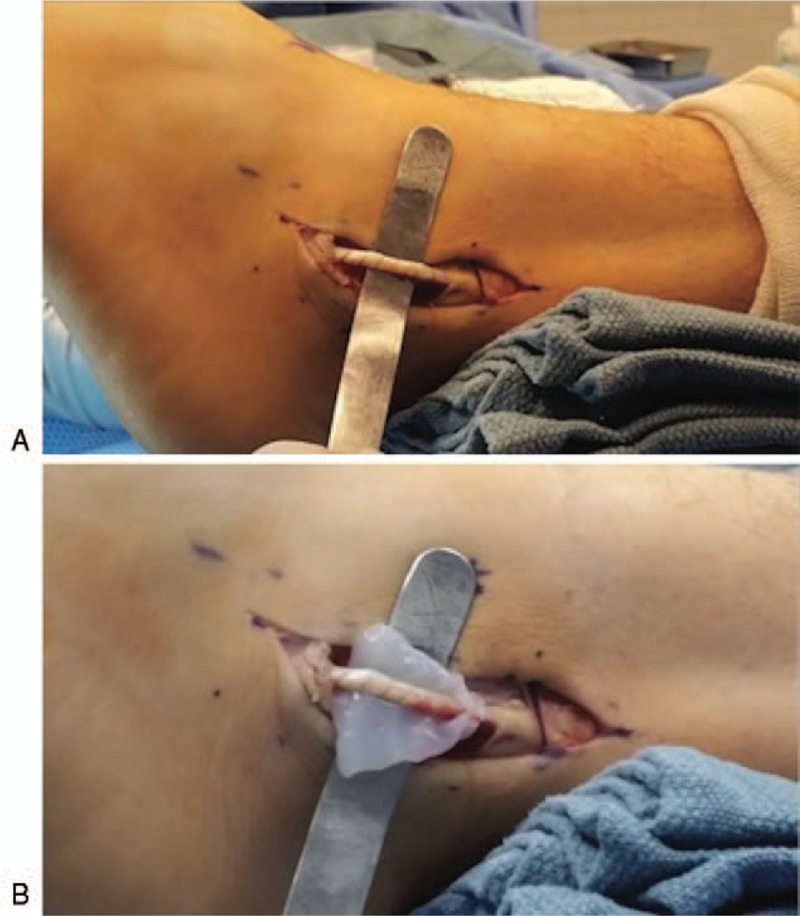
In the case of a peroneal tendon repair, the (A) repaired tubularized tendon is isolated and (B) a 3 x 6 centimeter vCUT is passed under the repaired tendon, and beginning with the proximal corners, is circumferentially sutured around the tendon of interest. vCUT= viable cryopreserved umbilical tissue.

The peroneus brevis tendon is isolated and the proximal and distal extents to the tear are identified. The portion of the tendon containing the tear is tubularized utilizing a #2 absorbable suture (Vicryl, Ethicon, Inc., NJ) in a continuous running suture type fashion (Fig. 2A). The area is flushed with copious amounts of sterile normal saline solution. At this time, a 3 x 6 cm vCUT graft is prepared and wrapped circumferentially around the tendon (Fig. 2B) and sutured in place utilizing a #3 absorbable suture (Vicryl, Ethicon, Inc., NJ).

**Figure 3:**
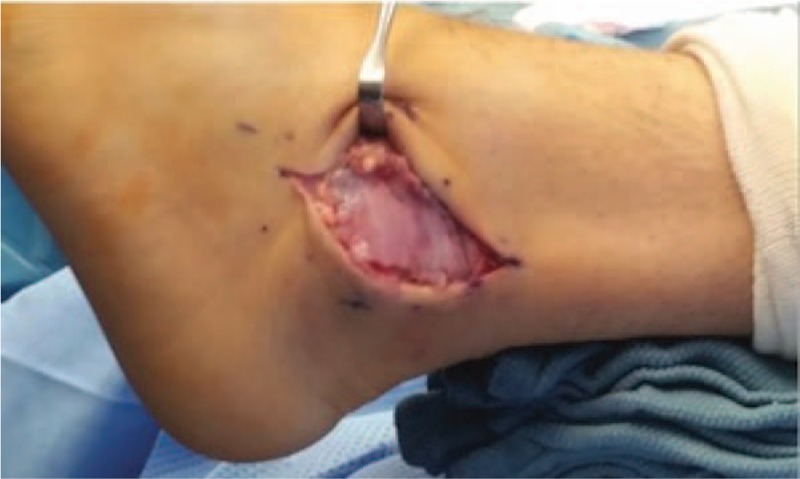
In a peroneal tendon repair, a second piece of 3 x 6 centimeter vCUT laid along the incision site before skin closure. vCUT= viable cryopreserved umbilical tissue.

Attention is then directed towards closure. A #3 absorbable suture (Vicryl, Ethicon, Inc., NJ), is used to close the peroneal tendon sheath and superior peroneal retinaculum in a continuous running suture type fashion. #4 absorbable retention sutures (Vicryl, Ethicon, Inc., NJ) are used to re-approximate the subcutaneous tissues in a simple interrupted suture type fashion. Here, a second piece of 3 × 6 cm vCUT is laid along the incision site (Fig. 3) before skin closure with a #5 absorbable suture in a horizontal mattress suture type fashion.

Before bandaging, a 10 mL injection consisting of 0.5% Marcaine plain is instilled proximal to the surgical sites. Attention is then directed towards bandaging consisting of non-adherent layer (Adaptic, Acelity, TX), 4 × 4 seconds, kling bandage roll, and kerlix gauze bandage roll applied in a mildly compressive nature. The pneumatic ankle tourniquet is rapidly deflated and instantaneous capillary refill is noted on the patient's digits. A below knee cast is applied to the lower extremity with the foot in dorsiflexion and slight eversion. The patient is discharged home with instructions to follow-up in 1 week.

## 3 Results

Patient demographics and pilot study endpoint outcomes are shown in Table 1. All 5 patients at 1-week post-op showed no visual signs of erythema, tenderness, heat and/or swelling and reported no narcotic use. Forty percent of patients (2/5) reported no pain at 1-week post-op. All patients were followed for an average of 24.15 months (range 16.75–26.5 months) after surgery. In that time, no post-op adverse events including, but not limited to, dehiscence, confirmed infection, fluid collection and/or drainage, were observed. Analysis of additional secondary investigative clinical outcomes demonstrated a reduction of pain in all 5 patients (average pre-op pain 8/10; average 1-week post-op pain 1.4/10). Average transition time in all patients to CAM boot and normal shoe was 3.2 weeks (range 2–4 weeks) and 5.6 weeks (range 4–8 weeks), respectively.

## 4 Discussion

Several surgical procedures to repair tendon ruptures are reported in the literature, yet no clear consensus on the best technique or rehabilitative protocol exists.^[1,3,11]^ Based on the severity of peroneal tendon rupture (e.g., partial-thickness split versus full-thickness split), treatment protocols vary from conservative treatment—including rest, ice, compression, and elevation—to invasive or percutaneous surgical repair.^[12]^ While surgical repair has proven to be effective, it has been reported that as many as 25.4% of patients struggle with post-op complications such as pain, and 17.4% experience persistent swelling.^[2]^ The optimal surgical technique would be one that prevents complications effectively and allows patients to resume normal activities in a reduced amount of time.

In current literature, few cases of acellular allograft use in tendon repair exist, yet reports detailing the surgical technique, safety, and outcomes with cellular placental allografts are scarce.^[13,14]^ The benefits of placental tissue use in surgery were initially described in ophthalmology in 1940 by DeRoth, and since then, more reports have surfaced.^[15–18]^ Specifically in the operative reconstruction of ocular surfaces, placental tissues have been shown to assist in minimizing post-op inflammation, pain, and adhesion formation.^[16,18]^ In response to such results seen in ophthalmology, Liu et al suggested that exploration of amniotic membrane application in other medical fields is warranted to explore potential benefit.^[17]^ Placental membrane use has since expanded to fields such as orthopedic surgery as an adhesion barrier and maxillofacial surgery for root coverage and reconstruction of temporomandibular joint ankyloses due to its antifibrotic properties.^[19,20]^ Based on the previous aforementioned outcomes of acellular placental tissue use in ophtlamology, orthopedic, and maxillofacial surgery, the authors wanted to explore the safety and possible clinical outcomes that a viable placental graft may offer to podiatric surgery.

According to the manufacturer, vCUT can be used as a tendon wrap in surgical tendon repair.^[21]^ Originating from human placental tissues, vCUT is aseptically processed from the umbilical cord, a structure composed of a 3-D extracellular matrix comprised of hyaluronic acid and collagen, growth factors, and viable cells including epithelial cells, fibroblasts and mesenchymal stem cells, and serves as a wrap to protect arteries and veins providing nutrients and gas exchange from the mother to the fetus.^[22]^ Due to the human umbilical cord's primary protective function and immuno-privileged nature, in theory, a product derived from umbilical or placental tissue and following FDA Guidance on homologous use,^[23]^ should pose no safety concern. With patient follow-up extending to over 2 years for the patient cohort presented, and the presence of no adverse events, the authors are confident regarding safe use of vCUT in patient surgery.

With safety confirmed, attention was drawn to secondary investigative clinical and rehabilitative endpoints demonstrating promising results. As a result of decreased pain and inflammation seen in all patients, rapidly resuming pre-injury activities, such as a weight-bearing without pain, was noteworthy, with the average return-to-activity time in normal shoe at 5.6 ± 1.3 weeks, which compares favorably to 15.2 ± 1.7 weeks previously reported for chronic tendon injuries repaired with acellular dermal allograft.^[24]^ The 2 Achilles tendon repair cases showed faster recovery by the ability to graduate from a non-weight-bearing cast to a CAM boot at 2 to 3 weeks post-oply versus 4 to 6 weeks reported in the literature for open repair of Achilles.^[25,26]^ The 2 peroneal cases demonstrated quick transition to CAM boot at 3 to 4 weeks and ambulation in normal shoe at 6 weeks, versus the reported median immobilization values of 6 weeks after primary repair, 6.3 weeks post grafting, and 8 weeks post tendon end-to-end suturing, presented in a review of 49 analyzed studies in total.^[3]^ Upon follow-up, all patients have remained pain-free with full range of motion comparable to pre-injury status, allowing them to regain previous functional status and 100% muscle strength.

To date, the authors have incorporated vCUT as a tendon wrap in over 30 cases. Documented patient outcomes have consistently included decreased pain and reduced inflammation and fibrosis, which have ultimately led to expedited recovery to a pre-injury status. Notably, the expedited transition of patients and improved rehabilitative outcomes has allowed the center to alter their original surgical and rehabilitative protocol. Previous protocol at Macomb Foot, Ankle and Wound Care, before incorporation of vCUT in surgical tendon repair, included keeping patients non-weight bearing in a cast for 3 weeks, followed by transition into a weight bearing cast for an additional 3 weeks, followed by subsequent transition, as tolerated, to CAM boot and normal shoe, with physical therapy initiation at 6 to 8 weeks. With the incorporation of vCUT, and successful management of post-op complications, patients are able to reduce rehabilitation time and return to pre-injury status faster by eliminating the need for 3 weeks weight-bearing cast and ability to immediately graduate from non-weight bearing cast to CAM boot with concomitant physical therapy.

Despite initially promising patient outcomes observed with utilization of vCUT in tendon repair, the authors recognize the limitations of this retrospective pilot evaluation. A prospective randomized study, comparing vCUT utilization in surgical tendon repair to non-augmented controls is necessary. Additional values such as daily recorded narcotic use and functional score evaluations both pre- and post-vCUT utilization in surgical tendon repair will offer additional insight. As a follow-up to this pilot study, the next step in the authors’ research is to confirm benefits of vCUT as a tendon wrap augmenting surgical tendon repair in a prospective case study. These findings are currently underway and plan to be reported on in a follow up publication.

## 5 Conclusion

Briefly, a surgical technique using vCUT allograft in peroneus brevis tendon repair is described. The authors have observed that the surgical technique is simple and safe for patients and preliminary findings have demonstrated favorable clinical and rehabilitative outcomes over historically observed controls, including minimal inflammation, pain, adhesion formation, and scarring with quick return of patients to pre-injury activities and lifestyle.

## Author contributions

**Conceptualization:** Elisabet K. Tassis.

**Data curation:** Kyle W. Sundblad.

**Investigation:** Kyle W. Sundblad, Elisabet K. Tassis.

**Methodology:** Kyle W. Sundblad, Elisabet K. Tassis.

**Software:** Elisabet K. Tassis.

**Supervision:** Kyle W. Sundblad.

**Visualization:** Kyle W. Sundblad, Elisabet K. Tassis.

**Writing – original draft:** Elisabet K. Tassis.

**Writing – review & editing:** Kyle W. Sundblad, Elisabet K. Tassis.
